# Rehabilitation of the P2X5 receptor: a re-evaluation of structure and function

**DOI:** 10.1007/s11302-022-09903-0

**Published:** 2022-10-24

**Authors:** Brian F. King

**Affiliations:** Research Department of Neuroscience, Pharmacology & Physiology (NPP), University College London (UCL), Gower Street, London, WC1E 6BT UK

**Keywords:** P2X receptor, P2X5 receptor, Ion channel, Purinergic, ATP

## Abstract

Of the extended family of ATP-gated P2X ion-channels, the P2X5 receptor has received comparatively little attention since first cloned over 25 years ago. Disinterest in studying this P2X subtype stems from two commonly held beliefs: (i) canonical human P2X5 is non-functional because the P2X5 subunit is truncated (hP2X5A, 422 aa) and missing the critical peptide sequence (22 aa) encoded by exon 10; (ii) rat and mouse P2X5 subunits are fully formed (455 aa) but the receptor is only weakly functional, and successive ATP responses rapidly run down in amplitude. However, newer studies have re-evaluated these notions. First, a low proportion (around 10%) of humans possess full-length P2X5 subunits (444 aa) and can form competent P2X5 receptors. Full-length P2X5 has been identified only in black Americans, but may occur in a wider population as more ethnicities are screened. Second, replacement of one of three amino acids in rat P2X5 subunits with corresponding residues in human P2X5 subunits (V67I, S191F, or F195H) significantly improves the responsiveness of rat P2X5 to ATP. Replaced residues exert an allosteric action on the left flipper, allowing the docking jaw for ATP to flex the lower body of the subunit and fully open the ion pore. This proposed action may drive the search for naturally occurring modulators which act allosterically on wildtype rat P2X5. This review collates the available information on the structure and function of human and rat P2X5 receptors, with the view to rehabilitating the reputation of these ATP-gated ion channels and stimulating future lines of research.

## Introduction

P2X receptors are membrane-inserted ion channels that are widely distributed among many cell types, where these ion channels are gated by extracellular ATP [1–3]. Seven human genes, *P2RX1-P2RX7*, encode peptide building blocks (the P2X subunits), which assemble into functional P2X receptors. The structure of human *P2RX* genes is complex, comprising either 11, 12, or 13 exons of varying sizes with intervening introns also of varying sizes and, accordingly, the complete genes vary considerably in size (12–40 kb) [1, 2]. The same is true for *P2rx* genes for mammalian and vertebrate species (*P2rx1–P2rx7*). Gene transcription may produce splice variants of the natural translation product, particularly when exon-skipping occurs. Notably, exon-skipping occurs in the transcription of the human gene *P2RX5*, which generates a non-functional isoform of the P2X5 subunit (see “hP2X5 receptor structure”). Nonetheless, a small subset of human subjects can form competent P2X5 receptors [1], and, to this end, the first half of this review re-evaluates the structure and function of competent human P2X5 receptors.

**Fig. 1:**
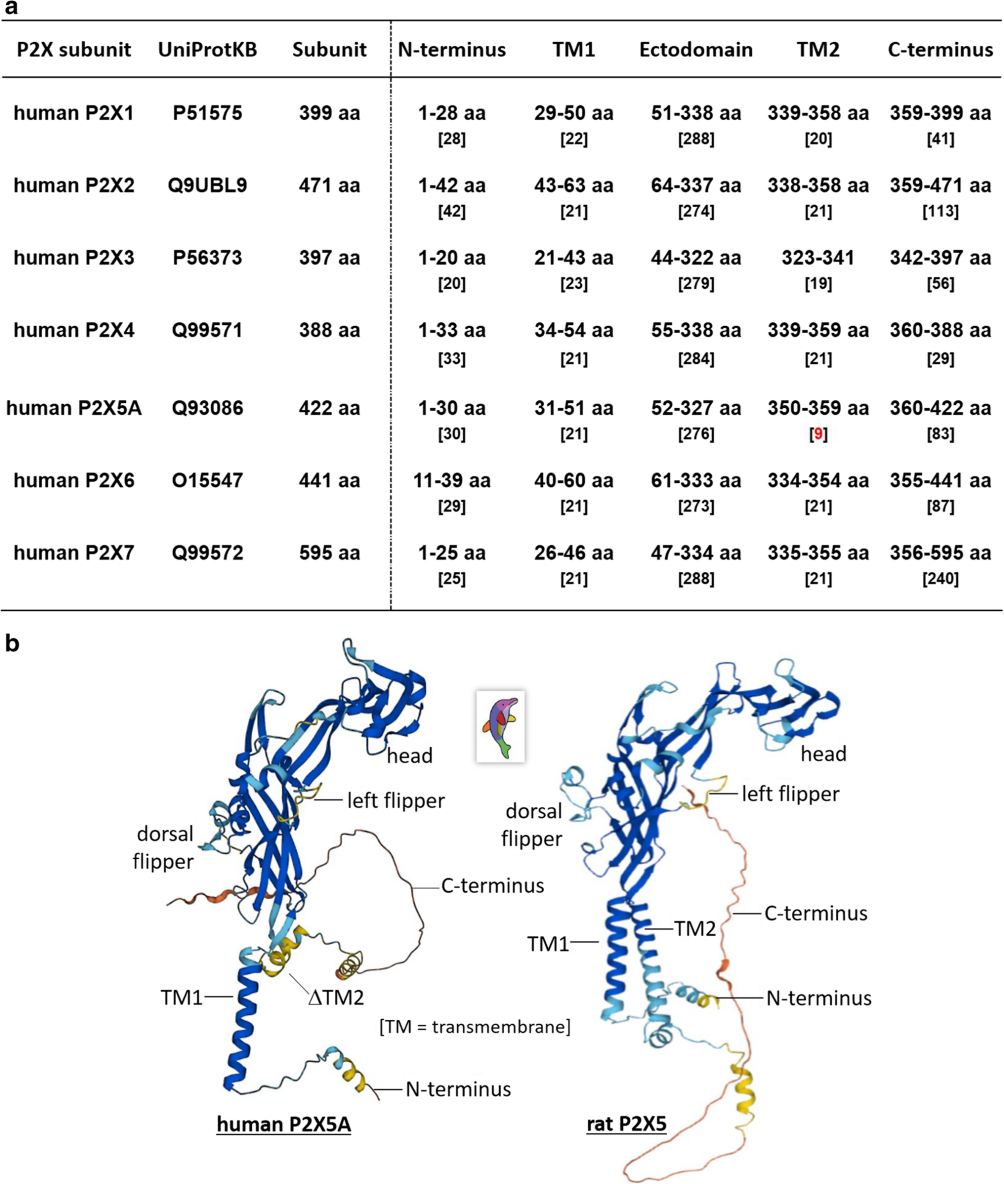
Topology of human P2X1–7 subunits. **a** Locations and length of peptide domains in the primary sequence of P2X1–7 subunit proteins. Data were obtained from the *UniProtKB* database. **b** 3D representation of the structure of the human P2X5A subunit (left) and rat P2X5 subunit (right) as predicted by *AlphaFold* [9], with inset showing the “dolphin” model of a P2X subunit [5]. *AlphaFold* produces a per-residue confidence score (pLDDT: local distance difference test) between 0 and 100. Model confidence: dark blue, pLDDT > 90; light blue, 90 > pLDDT > 70; yellow, 70 > pLDDT > 50; red, pLDDT < 50

Complete transcription and translation of *P2RX1–P2RX7* genes produce P2X subunits defined as P2X1–P2X7 proteins [1–3]. These subunit proteins range in length from 338 to 595 amino acids (aa) (see Fig. 1a). Evidence widely accrued from a range of studies (involving biochemical, biophysical, electron microscopic, pharmacological, and X-ray crystallography approaches) indicate that P2X receptor channels assemble as three P2X subunits [1–3]. P2X receptors can occur as homomeric assemblies of 3 identical P2X subunits (e.g. rat P2X5 receptors, rP2X5) but also can occur as heterotrimeric assemblies involving different P2X subunits (e.g. rat P2X1/5 receptors, involving rP2X1 and rP2X5 subunits) [1–3]. The many different heteromeric assemblies of mammalian P2X receptor subunits have been described in detail elsewhere [1–3].

**Fig. 2:**
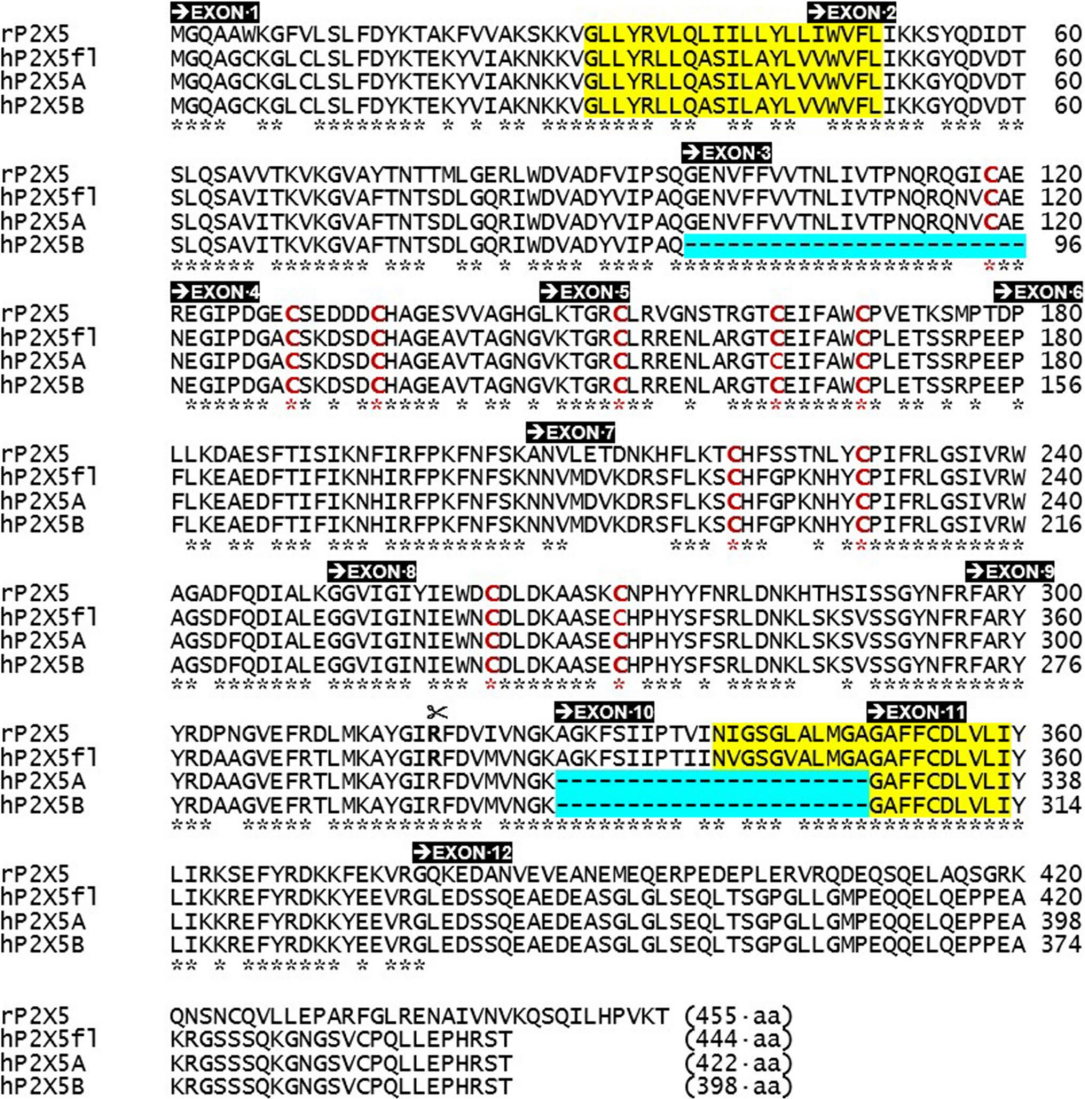
The amino acid sequence of P2X5 orthologues. The primary sequence of the rat P2X5 subunit protein (rP2X5, 455 aa), compared with the sequences of full-length human P2X5 subunit (hP2X5fl, 444 aa), isoform A of human P2X5 subunit (hP2X5A, 422 aa), and isoform B of human P2X5 subunit (hP2X5B, 398 aa). The positions of two transmembrane spanning regions (TM1 and TM2) are highlighted in yellow. The peptide sequences missing in hP2X5A and P2X5B are highlighted in blue. Exon 1–12 sequences are marked by black boxes. The scissors symbol identifies where the N-terminal region of hP2X5fl (1–318 aa) was spliced with the C-terminal of rP2X5 (318–455 aa) in [10]. The asterisks mark where amino acid residues are conserved between four sequences

**Fig. 3:**
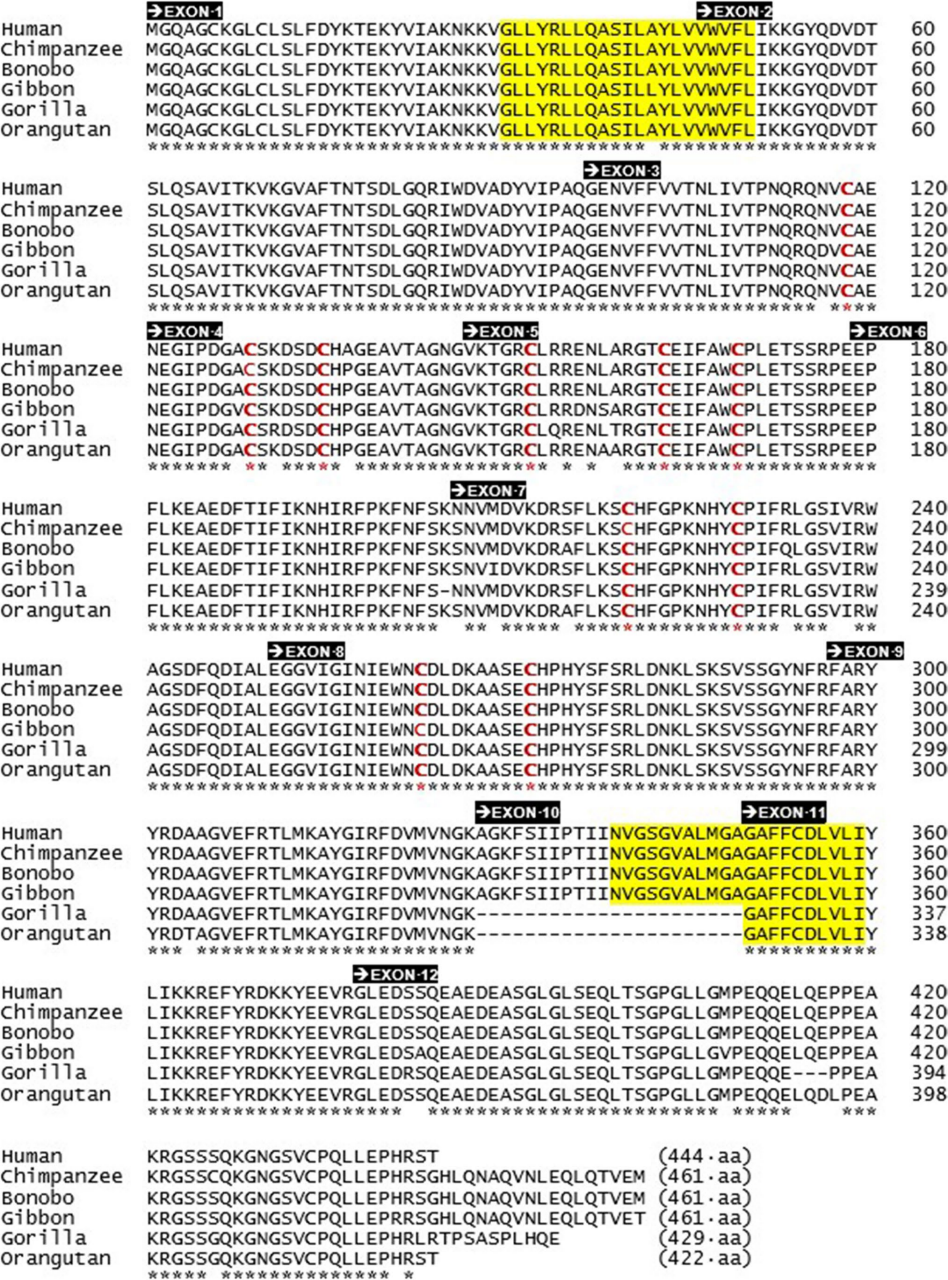
The amino acid sequences of P2X5 orthologues in *Hominoidea* species. The primary sequence of full-length human P2X5 subunit protein (*Homo sapiens*, 444 aa), chimpanzee P2X5 (*Pan troglodytes*, 461 aa), bonobo P2X5 (*Pan paniscus*, 461 aa), gibbon P2X5 (*Hylobates moloch*, 461 aa), gorilla P2X5 (*Gorilla gorilla*, 429 aa), and orangutan P2X5 (*Pongo abelii*, 422 aa). The positions of two transmembrane spanning regions (TM1 and TM2) are highlighted in yellow. Exon 1–12 sequences are marked by black boxes. The asterisks mark where amino acid residues are conserved between six sequences. Conserved cysteine residues are marked in red

**Fig. 4:**
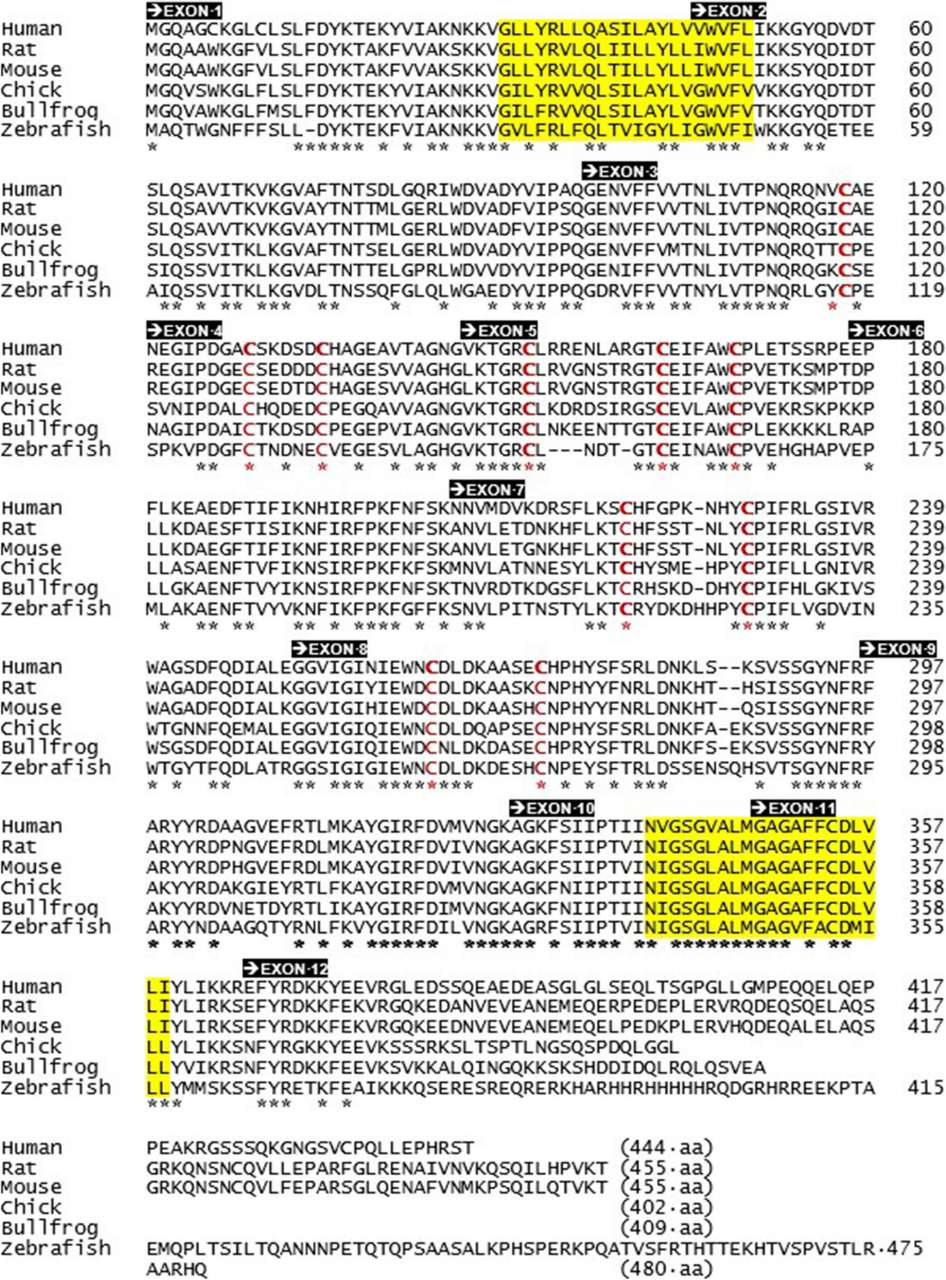
The amino acid sequences of P2X5 orthologues in vertebrate species. The primary sequence of full-length human P2X5 subunit protein (444 aa), rat P2X5 (*Rattus norvegicus*, 455 aa), mouse P2X5 (*Mus musculus*, 455 aa), chick P2X5 (*Gallus gallus*, 402 aa), frog P2X5 (*Rana catesbeiana*, 409 aa), and zebrafish P2X5 (*Danio rerio*, 480 aa). The positions of two transmembrane spanning regions (TM1 and TM2) are highlighted in yellow. Exon 1–12 sequences are marked by black boxes. The asterisks mark where amino acid residues are conserved between six sequences. Conserved cysteine residues are marked in red

P2X1–P2X7 subunit proteins share a common topology of intracellular N- and C-terminal domains and two transmembrane-spanning regions (TM1 and TM2), connected by a *N*-glycosylated large ectodomain [1–3]. The N-terminus sequences for human P2X subunits encoded by exon 1 are short in length (20 to 42 aa), whereas the C-terminus sequences for human P2X subunits (encoded by either exon 11 alone, or exons 11–12, or exons 11–13 in some cases), are highly variable in length (29–240 aa) [1]. The ectodomain and transmembrane sequences for human P2X subunits are encoded by exons 2–11 and are less variable in length, yet still long (274 to 288 aa). Sixty-eight residues are conserved across the seven P2X subunits including 12 glycine residues, 10 cysteine residues, and 6 lysine residues in the ectodomain [2, 4]. Based on the first described P2X crystal structure, the ectodomain contains four alpha helices (*a*2–5), 14 beta sheets (β1–14), and two glycans (g2 and g4), as well as the above mentioned and conserved 10 cysteine residues generating five disulphide bridges (SS1-5) to provide structural strength to the bulk of the P2X subunit [5]. Two short hydrophobic regions of the P2X subunit protein span the cell membrane as alpha helices (*a*-helices). The first *a*-helix (TM1) is 21–23 aa in length for human P2X subunits, and the second *a*-helix (TM2) is 19–21 aa in length ° except for the shorter TM2 sequence of human P2X5 where the exon 10-specified sequence is commonly missing. The sequence identity of human P2X1–7 subunit proteins ranges from 40.6 to 55.4%, for amino acid sequences covering the transmembrane spanning regions and ectodomain [2]. The variability in size and length of key peptide segments of human P2X1–P2X7 subunits is summarised in Fig. 1a, whereas exon boundaries for P2X5 isoforms are shown in Figs. 2, 3, and 4.

The P2X ion pore is lined principally by three angled TM2 helices aligned innermost in the channel, based on structural analysis of P2X crystals as well as functional studies using site-directed mutagenesis of the TM2 peptide [5–8]. The permeation path formed by three innermost TM2 helices is surrounded by three outermost TM1 helices, which serve a modulatory role in ion channel permeation [5–8]. Truncation of the TM2 helix, because of exon 10 skipping in the common isoform of human P2X5 receptors, leads to the misfolding of P2X5 subunits and their retention in the cell cytoplasm. 3D-representations of the misfolded human P2X5 subunit created by *AlphaFold* [9], alongside the correctly folded rP2X5 subunit, are illustrated in Fig. 1b. Significantly, splicing the exon 1–9 sequences of the hP2X5 subunit (isoform A, hP2X5A) with the exon 10–13 sequences of the rP2X5 subunit creates a chimeric mutant that is successfully trafficked to the cell surface membrane and can be activated by extracellular ATP [10]. The functionality of this chimeric human/rat P2X5 receptor indicates that ATP-binding pockets do occur naturally for human P2X5A subunits, in the inter-subunit spaces of a trimeric assembly and at a docking jaw formed by the head region and dorsal fin of adjacent subunits, at approximately 40 Å above the ion pore [1, 5, 6, 8].

Rat P2X5 subunits can form functional homomeric assemblies responsive to extracellular ATP [11], but the currents evoked by rP2X5 receptors are exceedingly small compared with currents evoked by other mammalian P2X receptors [e.g. see Fig. 2 in Ref 11]. The same is true for homomeric assemblies of mouse P2X5 subunits (mP2X5), the primary sequence of which shares 95% identity with the rP2X5 subunit [12]. Thus, rodent P2X5 receptors represent a perplexing conundrum where the TM2-lined permeation pathway is perfectly functional and yet the ATP-binding pockets of the ectodomains do not function well. These circumstances contrast vividly with human P2X5 receptors where the precise opposite occurs. To this end, the second half of this review re-evaluates the structure and function of rat P2X5 receptors.

The overall aim of this review is to rehabilitate the reputation of mammalian P2X5 receptors, which are generally viewed as inconsequential among the otherwise often-studied and extended family of P2X receptor subtypes. A fresh perspective on the functionality of P2X5 subtype represents a new dawn for the molecular physiology of ATP-gated ion channels and a new understanding of the human P2X5 receptor in health and disease.

## Human P2X5 receptors

### hP2X5 receptor structure

A cDNA for an incomplete P2X5 isoform (a subunit protein of 422 amino acids, 422 aa) was isolated from a cDNA library of human cerebellum [10]. This discovery followed the initial identification in the same study of a cDNA encoding an incomplete and shorter P2X5 isoform (398 aa subunit protein), isolated form a human foetal brain cDNA library scanned for sequences homologous to the entire coding region of the rP2X4 subunit [10]. Both these long and short P2X5 isoforms of the translated proteins came to be known as Isoform-A and Isoform-B, respectively [2]. Both are truncated sequences of the full-length human P2X5 subunit [10], because Isoform-A (hP2X5A, or hP2X5Δ^328–349^) lacks the 22 aa residues encoded by exon 10, whereas Isoform-B (hP2X5B, or hP2X5Δ^97–120/328–349^) lacks the 24 aa residues encoded by exon 3 in addition to lacking the 22 aa residues encoded by exon 10 [2, 10]. The primary amino acid sequence of these two truncated hP2X5 isoforms [10] are compared against the sequence of rP2X5 [11], as well as the full-length hP2X5 sequence (see Fig. 2). Critically, the missing exon 10 residues help to form part of the second transmembrane spanning region (TM2) of the P2X5 subunit. Consequently, an incomplete TM2 and the misfolding of truncated P2X5 subunits result in subunit aggregation and retention in the cell cytoplasm [13]. Progressive reintroduction of exon 10 residues in a series of hP2X5 constructs requires the minimum insertion of 11 residues (328–338) before trimeric assemblies are formed, and requires the full insertion of 22 residues (328–349) before functionality is observed [13].

The possibility of a full-length and functional human P2X5 receptor was first proposed after the accidental discovery of a complete P2X5 gene in human genomic fragments (e.g. Genbank: clone AF168787.1), in a study that paradoxically focussed on the isolation of the mouse P2X5 gene (*P2rx5*) [12]. Therefore, the human P2X5 gene is not a pseudogene but may be transcribed incompletely with exon 10 skipped. The full peptide sequence encoded by exon 10 was identified as *Ala-Gly-Lys-Phe-Ser-Ile-Ile-Pro-Thr-****Ile****-Ile-Asn-****Val****-Gly-Ser-Gly-****Val****-Ala-Leu-Met-Gly-Ala*, which shows three conservative differences (I337V, V340I, V344L) compared with the rat and mouse P2X5 protein sequences [12]. Soon afterwards, the human exon 10 sequence was confirmed by a second research group when probing human genomic DNA with the exon 10 cDNA sequence for rP2X5 [14]. A full-length construct of human P2X5 was prepared for expression studies and the resultant full-length protein (444 aa subunit) assembled into a functional hP2X5 receptor [14, and then see 15–18]. However, the primary sequence of the full-length human P2X5 protein (hP2X5-fl) has never been listed in popular databases (such as *Ensembl* and *UniportKB*). Accordingly, isoform A (hP2X5A, 422 aa) is routinely identified in these databases as the canonical P2X5 isoform, even though this isoform cannot give rise to functional receptors. Another 10 non-functional splice variants appear in the *Ensembl* database, but hP2X5-fl does not. For the sake of clarity and fullness, the primary sequence of the hP2X5-fl protein is shown in Fig. 2.

**Fig. 5:**
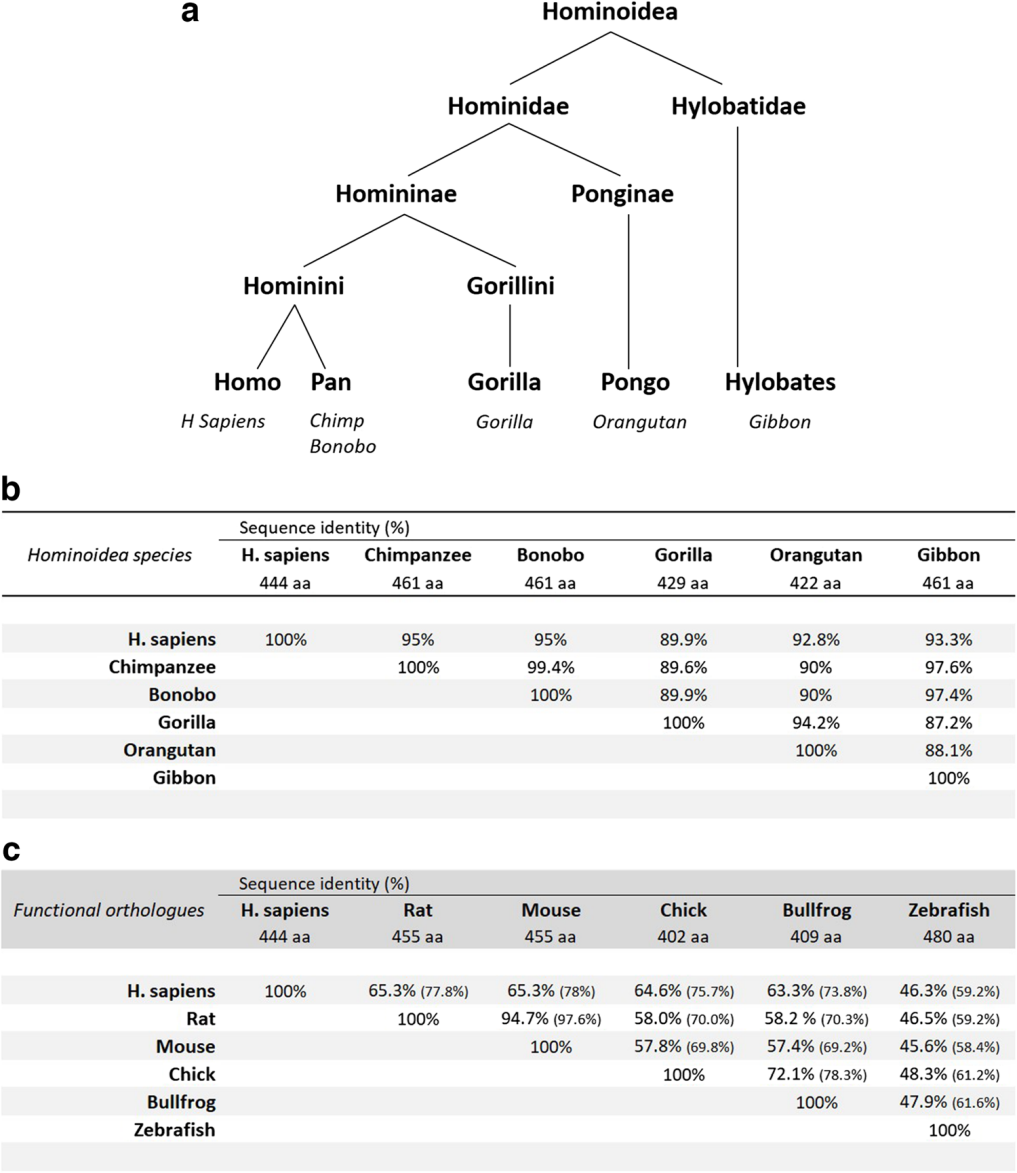
Sequence identity of P2X5 orthologues. **a** Cladistic relationship between extant hominoid species (cladogram). Humans (*Homo sapiens*) are most related to *Pan* species. **b** Sequency identity of P2X5 subunits in six *Hominoidea* species, including humans. **c** Sequence identity of P2X5 subunits in six vertebrate species, including humans, which possess functional P2X5 orthologues. In brackets, sequence identity of proteins encoded by exons 1-11

The full-length human and rat P2X5 protein sequences are highly related (78% identical up to the end of exon 11), and then diverge after Arg^377^ at which point the exon 12 sequence begins. Considerable divergence from exon 12 onwards results in a lower overall identity of 65% for the full-length human and rat proteins (see Figs. 2 and 5c). Nonetheless, the C-terminus sequence of the human P2X5 subunit is highly conserved in five extant hominoid species (bonobo, chimpanzee, gibbon, gorilla, and orangutan; see Fig. 3). The primary sequence of all hominoid C-termini differs substantially from the C-termini of rat and mouse P2X5, an outcome postulated to be the result of gene splicing in an ancient *hominoidea* ancestor [12]. Future anthropological studies may eventually identify that early hominoid ancestor.

Truncation of the P2X5 cDNA to remove the bulk of intracellular C-terminus beyond Arg^337^ still results in functional rat and human P2X5 receptors, although the amplitude of maximally-evoked currents is reduced without changing any other pharmacological or operational property [14]. Thus, the peptide sequence encoded by exons 12 and 13 in rat and human P2X5 are not obligatory for functionality. For hP2X5, the intensity of fluorescence for P2X5 subunit proteins in the cell membrane of HEK293 cells transfected with C-terminal truncated hP2X5 labelled with enhanced green fluorescent protein (hP2X5ΔC-EGFP) appears to be similar to the intensity of fluorescence for membrane-inserted full-length hP2X5-EGFP [see Fig. 1c in Ref 14]. Thus, it seems unlikely that the reduced maximal currents for hP2X5ΔC were due to a lower level of receptor trafficking to the membrane. Since unitary conductance for full length and truncated hP2X5 receptors was not measured [14], it remains to be shown why removing the bulk of the intercellular C-terminus results in reduced maximally-evoked currents.

Further progress has been made in uncovering a natural source of the human P2X5-fl receptor, through genotyping 76 human DNA samples taken from different ethnic cohorts in the USA: white American, black American, Middle Eastern, and Chinese donors [15]. Of these cohorts, black Americans (7 of 10 subjects) express the full-length P2X5 protein whereas the other cohorts (white American, 46 of 46; middle Eastern ethnicity, 10 of 10; Chinese ethnicity, 10 of 10 subjects) express the truncated P2X5 protein (hP2X5A, hP2X5Δ^328−349^). It has been argued that a single-nucleotide polymorphism (SNP) can occur at the 3′-splice site of exon 10 of the human P2X5 gene, replacing a critical thymidine (T) with a guanine (G) nucleobase [13–16, 18]. The G-allele results in splice-skipping of exon 10, leading to truncated P2X5 subunits in those human cDNA samples tested so far (69 of 76 subjects; 91%) [15, 16]. However, the T-allele results in full-length P2X5 subunits that assemble into functional P2X5 ion channels, which were confirmed in human lymphocytes harvested from black Americans possessing the T-allele [15]. By crude analysis, approximately 9% of human genomic DNA samples possess a competent P2X5 gene [15]. The mature human P2X5-fl is 444 aa in length and, thus far, has only been found in a limited number of human cDNA samples tested but also in a limited number of ethnicities tested.

Transcripts encoding the full-length P2X5 receptor have been reported in some, but not all, *Hominoidea* species (Fig. 3). Clearly, P2X5-fl is expressed in some humans, but also occurs in chimpanzee (*Pan troglodytes*), bonobo (*Pan paniscus*), and gibbon (*Hylobates moloch*) where the exon 10 peptide is identical and their intracellular C-termini are highly similar in these hominid species (Figs. 3 and 5b). A truncated P2X5A isoform has been reported in the *Ensembl* database for cDNA libraries of gorilla (*Gorilla gorilla*) and orangutan (*Pongo abelii*). Perplexingly, Kotnis and colleagues have reported that the exon 10 sequence is present in cDNA libraries for gorilla (*Gorilla gorilla*) and orangutan (*Pongo pygmaeus*) [15]. This apparent contradiction probably indicates that complete P2X5 transcription may occur in some subspecies of these two hominids, whereas the same SNP as occurring in humans may occur in other hominid subspecies.

The possibility exists that one or more cell types from *Hominoidea* species can serve as surrogates to study the equivalent human P2X5 receptors. The closest *Hominini* ancestors to modern man are represented by the *Pan* species, which include chimpanzee and bonobo (see Fig. 5a, b). The full-length human P2X5 subunit shows a 95% sequence identity to the P2X subunits of these close ancestors (see Fig. 5b). The P2X5 subunit of the most distant *Hominoidea* species (gibbon) also contains an exon 10 sequence and is 93% identical with human P2X5 (see Fig. 5b). Otherwise, P2X5 subunits of *Hominidae* ancestors (gorilla and orangutan) are 90–93% identical, yet non-functional when missing the exon 10 sequence (Fig. 5b).

Hominid lymphocytes may represent a useful cell type as a surrogate in the study human P2X5 receptors. Human P2X5 subunits are highly expressed in these immune cells [10] and are highly prevalent in a broad range of lymphoid malignancies: T and B cell acute lymphoblastic leukaemia, chronic lymphocytic leukaemia, B cell lymphomas, and multiple myelomas [19, 20]. Kotnis and colleagues collected human lymphocytes when genotyping American ethnic groups for truncated and full-length P2X5 subunits [15]. P2X5 subunits are prevalent in naïve and memory B-cells, among the many tissues sampled so far for the Human Protein Atlas (see https://www.proteinatlas.org/search/P2X5). For African American subjects, their lymphocytes may represent a useful cell type for the purpose of cloning and confirming the primary sequence of the naturally-occurring and full length P2X5 protein. However, relatively few subjects from other ethnicities have been tested for hP2X5-fl and a wider gene screening programme may be required.

### hP2X5 receptor function

The number of functional studies of hP2X5-fl is limited, mostly because a functional receptor was not thought to exist in humans. Also, the cDNA for hP2X5-fl was not readily available and each laboratory was required to generate a construct with the exon 10 coding region correctly inserted into the cDNA of canonical hP2X5A. So far, only four laboratories have succeeded in generating this construct and then characterised the resultant full length hP2X5 receptors [see 13, 15–18]. The results of these functional studies are summarised in Table 1.

**Table 1:** Operational and pharmacological profiles of hP2X5-fl receptors

hP2X5-fl	[Ref. 14] (2003)	[Ref. 13] (2006)	[Ref. 15] (2010)	[Ref. 16] (2019)	[Ref. 17] (2021)	[Ref. 18] (2022)
ATP responses						
* Expression system*	HEK293 cells	*X. laevis* oocytes	1321N1 cells	HEK293 cells	1321N1 cells	*X. laevis* oocytes
* Activation*	fast (< 500 ms)	fast	fast	fast	fast	fast (< 300 ms)
* Inactivation*	slow (> 5 s)	biphasic	slow (> 2 s)	slow (> 5 s)	slow (> 5 s)	slow (> 5 s)
* P*_*Ca*_*/P*_*Na*_	1.5	^___^	^___^	^___^	Ca^2+^ permeable	^___^
* P*_*Cl*_*/P*_*Na*_	0.5	^___^	^___^	^___^		Cl^−^ permeable
Agonist EC_50_ values						
* ATP*	4.1 ± 0.5 μM	^___^	0.3 μM	2 μM	1 μM	39 μM
* BzATP*	5.7 ± 0.9 μM	^___^	^___^	^___^	^___^	~ 10 μM
*α,βmeATP*	161 ± 35 μM	^___^	12.2 μM	^___^	^___^	^___^
Antagonist IC_50_ values						
* PPADS*	0.2 ± 0.02 μM	^___^	0.65 μM	^___^	7.0 μM	^___^
* Brilliant blue G*	0.53 ± 0.04 μM	^___^	^___^	^___^	^___^	^___^
* Suramin*	2.9 ± 0.4 μM	^___^	13 μM	^___^	16 μM	^___^
* TNP-ATP*	[10% at 1 μM]	^___^	2.5 μM	^___^	^___^	^___^
Allosteric modulators						
* nimodipine*	^___^	^___^	^___^	^___^	^___^	PAM (3–30 μM)
* amlodipine*	^___^	^___^	^___^	^___^	^___^	NAM (> 30 μM)

The most complete characterisation of hP2X5-fl was made by Alan North and his colleagues who concluded that its operational profile was similar to rP2X2 receptors [14]. Extracellular ATP (1–100 µM) elicited robust inward currents (1–5 nA, at − 60 mV) when hP2X5-fl was expressed heterologously in HEK293 cells [14], and where evoked responses were similar in amplitude and time-course to ATP responses for hP2X2 receptors [for example, see Fig. 7 in Ref 21]. Also, ATP-activated hP2X5-fl produced robust inward currents (1–5 nA, at − 40 mV) when expressed in 1321N1 astrocytoma cells [15] and, additionally, produced strong calcium signals that were similar in amplitude and time-course to ATP-activated hP2X2 receptors expressed in 1321N1 cells [17]. Where *Xenopus* oocytes have been used to express hP2X5, high concentrations of extracellular ATP (1 mM) elicited large inward currents (~ 5 μA, at − 60 mV) which inactivated in a biphasic manner, with a first phase decaying over several seconds followed by a second phase of inactivation lasting 10–30 s [13]. A related and earlier study has shown that the inactivation trajectory of some P2X receptors (notably rP2X2) varies according to the ATP concentration used, and may appear as biphasic in either oocytes or HEK293 cells when high ATP concentrations are used [22]. Therefore, differences observed in the inactivation rate of ATP responses at hP2X5-fl are not without precedence for the P2X2 subtype. These findings generally support the conclusion that hP2X5-fl responses do look like P2X2 responses [14]. There is one fundamental difference between ATP-activated hP2X5-fl and hP2X2 receptors; based on anion substitution experiments, the former has been shown to be permeable to chloride ions [14, 18], whereas the latter is not [14].

Apart from ATP, hP2X5-fl can be activated by either BzATP or *α*,βmeATP with an agonist potency order of ATP > BzATP ≫ α,βmeATP (see Table 1). Human P2X5-fl also is activated by 2-MeSATP although its agonist potency was not determined, whereas ATP γ S, ADP, and β, γ meATP were reported to be partial agonists [14]. Other studies have confirmed some of these pharmacological data, showing that α,βmeATP [13] and BzATP [18] are agonists at hP2X5-fl, yet less potent than ATP (see Table 1). Since hP2X5-fl responses are similar in appearance to hP2X2 responses, it is important to be aware of their respective agonist profiles. For hP2X2, an agonist potency order of BzATP > 2-MeSATP > ATP > ATP γS was reported for a calcium-influx assay [23]. Notably, α,βmeATP and ADP did not show agonist activity at hP2X2 at concentrations up to 100 µM [23]. North and colleagues also reported that α,βmeATP is much more potent at hP2X5-fl than at rP2X2 receptors [14]. Therefore, the operational profile of hP2X5-fl is a slowly desensitizing, α,βmeATP-sensitive P2X channel permeable to sodium, potassium, calcium, and chloride ions, but similar only in some ways to the operational and pharmacological profiles of hP2X2 receptors. The same conclusions were drawn for a comparison of rP2X5 with rP2X2 receptors (see “rP2X5 receptor function (1996 to 2005)”).

In antagonist studies, ATP responses at hP2X5-fl were blocked by PPADS, Brilliant blue G (BBG), suramin, and TNP-ATP (see Table 1). The potency order of BGG > PPADS > suramin is unremarkable, since all of these compounds are considered to be nonselective antagonists for P2X receptors [1]. Other compounds are known to be highly-potent and relatively selective antagonists of P2X2 receptors: for example, NF770 [24], PBS-1011, and PBS-10211 [25]. Their comparative activity has been reported for rP2X1, rP2X3, rP2X4, and rP2X7 receptors, but they remain to be tested on ATP-activated hP2X5-fl or rP2X5 receptors [26]. To date, there are no antagonists that selectively discriminate between hP2X5-fl and the other P2X receptor subtypes.

A recent study has shown that hP2X5-fl can be modulated by 1,4-dihydropyridines (DHPs), with nimodipine acting as a positive allosteric modulator (PAM) and amlodipine acting as a negative allosteric modulator (NAM) [18]. The precise mode of action of DHPs at hP2X5-fl is unknown, but has been assumed to be allosteric because it requires the continued presence of both agonist and modulator [18]. Both of these DHP compounds are better known as L-type calcium channel blockers and are used commonly in the clinic for cardiovascular disorders. Nimodipine is prescribed for delayed cerebral ischaemia following subarachnoid haemorrhage [27], whereas amlodipine is used as a once-daily oral antihypertensive agent particularly in the aged patient [28]. However, there have been no focussed studies on the side-effects of these L-type calcium channel blockers in patients of American Black origin and where the possibility of functional P2X5 receptors has to be taken into account. Other DHPs are known to interact with P2X receptors, notably nicardipine which inhibits ATP responses at rP2X2 and rP2X4 receptors [29]. Additionally, 5-phosphonate derivatives of 1,4-DHPs (MRS2154 and MRS2155) are PAMs for rP2X2 receptors [29]. These compounds and more need to be tested on hP2X5-fl receptors, to elevate our understanding of this subtype to the same level as found in the latest IUPHAR review for the operational profiles of homomultimeric P2X1-4 and P2X7 receptors [1].

## Rat P2X5 receptors

### rP2X5 receptor structure

A cDNA for rP2X5 was amplified from 440 bp PCR fragments of mRNAs extracted from rat coeliac and superior mesenteric ganglia (containing sympathetic neurons that innervate the upper GI tract, kidney, liver, pancreas, and adrenal glands) [11]. Shortly thereafter, an identical cDNA was isolated by PCR amplification of mRNA extracted from rat heart [30]. A third P2X5 cDNA was isolated from mRNAs extracted from rat spinal cord, although this cDNA does not appear in common databases [31]. The primary sequence of the rP2X5 subunit is 455 aa in length and is shown here alongside other functional P2X5 receptor isoforms in vertebrate species (Fig. 4).

The full-length rP2X5 subunit protein shares 95% sequence identity with the mouse P2X5 subunit (mP2X5), or 98% for the exon 1–11 sequences (see Figs. 4 and 5c), bearing in mind that rP2X5 remains functional when truncated at the end of the exon 11 sequence [14]. Both rat and mouse P2X5 receptors are functional as homomeric assemblies; however, they share the same characteristic of producing relatively small currents when activated by extracellular ATP [1, 2, 11, 12, 14]. These ATP responses were at least 10–100 fold smaller than those evoked by other recombinant P2X receptors under the same conditions (e.g. see Fig. 2 in Ref 11). Similar small ATP responses were reported for homomeric assemblies of zebrafish P2X5 subunits (zP2X5) [32], which since has been renamed zP2X5.1_tv1_ (transcript variant 1) [33]. However, a second transcript variant zP2X5.1_tv2_ was subsequently identified and shown to respond to extracellular ATP with large biphasic inward currents [33]. The zP2X5.1_tv1_ variant differs by 9 amino acids from zP2X5.1_tv2_ (G39V, H116Y, L144H, Y283S, T284Q, A285H, L286S, G287V, P288T) [33]. The peptide sequence of zP2X5.1_tv2_ is shown in Fig. 4 and shares 46% sequence identity with rP2X5 and mP2X5 proteins (Fig. 5c). Chick and bullfrog isoforms of P2X5 receptors also elicited large responses to extracellular ATP [34–36], and their P2X5 subunit proteins share 58% sequence identity with full-length rP2X5 and mP2X5 proteins (Fig. 4c). As an aside, chicken P2X5 was first identified as chick P2X8 [34] but is now recognized as P2X5 receptor [35]. Similarly, frog P2X5 was first identified as bullfrog P2X8 [36] but is also accepted as part of the P2X5 family [33].

A C-terminus motif Y*XXX*K is known to regulate the trafficking and surface expression of competent ATP-gated P2X channels [37]. This motif occurs eight residues downstream of the end of the second transmembrane domain for rP2X1-6 (with a frameshift of a further 18 aa downstream for rP2X7), and is present in rP2X5 subunits as well as other species isoforms of P2X5 (Figs. 2, 3, and 4). Single-point mutation of the tyrosine (Y) or lysine (K) residues to alanine (A) greatly decreased the surface expression of rP2X5 [37]. However, the Y*XXX*K motif (368–372) is naturally present in wildtype rP2X5 receptors, as well as in mutant rP2X5 receptors truncated at the end of exon 11 (rP2X5-Δ^378–455^), suggesting that their small ATP responses were not primarily due their level of surface expression but attributable instead to other factors.

Functional homomeric P2X receptors (P2X1-5 and P2X7) are modulated by membrane phospholipids, notably PIP_2_ (phosphatidylinositol 4,5-biphosphate) [38]. For inside-out membrane macropatches expressing rP2X5 receptors, the superfusion of a soluble PIP_2_ analogue prevented the rundown of inward currents evoked by extracellular ATP in the patch pipette and further activated these ATP-gated ion channels; both effects were neutralised by polylysine (polyK), which binds to and sequesters free PIP_2_ [38]. Naturally occurring PIP_2_ accounts for only 1–3% of the total cellular lipid content of the cell membrane, but it is concentrated in the inner leaflet of the plasmalemma where its negatively charged headgroup can interact electrostatically with positively charged aa residues at the juxtamembrane region of the C-termini of ion channel subunits [39]. A phosphoinositide-binding motif involving two short clusters of positively charged amino acids has been identified in the juxtamembrane region of C-termini for P2X1,2,4 subunits (but not in P2X3 and P2X7 subunits, even though their receptors are potentiated by PIP_2_ by another, yet unknown, mechanism) [40]. The first cluster in wildtype rP2X5 subunits has a lower complement of lysine (K) residues, and the insertion of positively charged amino acids (either S365K-E366Y or S365K-E366Y-E374K) creates rP2X5 mutants which acquired de novo PIP_2_ regulation when expressed in oocytes and also greatly increased the amplitude of their ATP-evoked currents (from 20 nA to 3 μA) [40]. Enhanced ATP responses at these rP2X5 mutants were inhibited by wortmannin, a nonselective inhibitor of PI-kinases involved in PIP_2_ production, and wortmannin also reduced ATP responses at hP2X5 which possesses a more competent PIP_2_ binding motif [40]. Thus, an argument can be made that relatively small differences in the juxtamembrane C-terminal sequence can account partly for the high degree of variability of P2X5 phenotypes.

Apart from phospholipid regulation of channel function, P2X5 receptors also are affected by Ca^2+^-influx during long-lasting (> 15 s) channel activation by ATP. One form of Ca^2+^-dependence was described first for chick P2X5 [34], then frog P2X5 [36], and thereafter for zfP2X5 (variant 2) [33]. In these cases, a reduction of extracellular Ca^2+^ (from 1.8 to 0.3 mM, cP2X5 oocyte expression [34]; from 1.8 to 0 mM, fP2X5 oocyte expression [36]; from 2 to 0 mM, zfP2X5_tv2_ oocyte expression) changed ATP responses from a monophasic to a biphasic inward current (*I*_1_ and *I*_2_), where the second phase was considerably larger in amplitude and much slower than the first (*I*_2_ ≫ *I*_1_). These biphasic ATP responses for cP2X5 bear a striking resemblance to biphasic ATP responses produced by rP2X4 (expressed in either oocytes or HEK293 cells), where the *I*_2_ response of the latter also is inhibited by raising the level of extracellular Ca^2+^ from 0 to 5 mM [41]. The transition towards the *I*_2_ state of ATP-evoked currents recorded under bi-ionic conditions is known to be accompanied by time-dependent alterations in the concentration of channel-permeant ions inside the cell, a shift in the reversal potential to less negative voltages, and a decrease in the slope of the current–voltage (*I*-*V*) relationship [42]. A further study has shown that the *I*_2_ transition is not due to a time-dependent progressive dilatation of the P2X ion pore, although pore dilation may still occur but through a regulatory element external to the P2X pore [43].

The ion pore of all P2X receptor subtypes (including rP2X5) shows a conserved glycine (G) residue in the centre of the TM2 region [4, 7]. Glycine can provide structural flexibility to peptide sequences and often is found close to the narrowest part of an ion pore [41]. Replacement of the glycine residue in rP2X4 with the large nonpolar tyrosine residue (G347Y) abolished the *I*_2_ response [41]. Conversely, replacement of the same residue in rP2X4 with positively charged residues (G347K and G347R) greatly diminished the amplitude of the *I*_1_ response [41]. However, biphasic responses have never been reported for rP2X5, and none of these single substitutions have ever been made in the primary sequence of rP2X5 subunits.

A second form of Ca^2+^ dependence also was described for chick P2X5 [34, 35] and rat P2X5 [44]. The amplitude of ATP responses at cP2X5 expressed in HEK293 cells showed a considerable degree of rundown, with a time-constant of 3.5 min, to repeated applications of ATP at submaximal concentrations in the presence of extracellular calcium (1.5 mM) [35]. In similar experiments with cP2X5 in oocytes, ATP responses were only 7% of the initial amplitude at 5 min after the removal of ATP, 31% at 30 min, and 65% at 1 h in the presence of extracellular Ca^2+^ (1.8 mM) [34]. Rundown of ATP responses also occurs with rP2X5 expressed in oocytes, with a time-constant of approximately 25 min in the presence of extracellular calcium (1.8 mM) [44]. Rundown is ameliorated for cP2X5 in low extracellular calcium (< 0.5 mM), and was prevented completely for rP2X5 when extracellular Ca^2+^ was replaced with extracellular Ba^2+^ during the recovery period from agonist responses and then returned shortly before the next agonist application [44]. The precise mechanism for rundown with either cP2X5 or rP2X5 has yet to be explained, although some attention was first given to a conserved consensus site (^18^T*X*[K/R]^20^) for PKC recognition in the intracellular N-terminus of all P2X subunits [44–46]. Replacement of the threonine phospho-acceptor in the T*X*K/R motif affects the desensitisation rate of some P2X receptors [45, 46], albeit in an inconsistent manner between P2X receptor subtypes [46] and, ultimately, through an action on the expression system and not directly on P2X receptors [46]. Otherwise, the intracellular C-terminus can affect the desensitisation rate of some P2X receptors (notably P2X2, where there are two threonine phospho-acceptors) [45]. However, rP2X5 lacks a threonine acceptor on its C-terminus (see Figs. 2 and 4). Other studies have shown that Ca^2+^-dependent desensitisation is a widespread phenomenon among many of the P2X receptor subtypes, the presumed locus for which is a high-affinity Ca^2+^ microdomain that also is affected by intracellular ATP and the metabolic status of P2X-expressing cells [47, 48]. Thus far, the rundown of ATP responses at rP2X5 receptors is poorly understood, partly through the absence of a unifying hypothesis for the desensitisation process of P2X subtypes, and partly because there is limited interest in studying rP2X5 itself.

### rP2X5 receptor function (1996 to 2005)

In the first 10 years following the cloning of rP2X5 (in 1996), only a few studies were successful in characterising homomeric rP2X5 receptors in any detail [11, 30, 44], whereas other studies were hampered by small ATP responses as well as Ca^2+^-dependent rundown of these small ATP responses [31, 49, 50]. The available information is summarised in Table 2. The extended pharmacological profiles from three successful studies are similar, confirming that rP2X5 was activated foremost by the non-hydrolysable nucleotide, ATP γS (0.03–300 µM), with the synthetic nucleotide 2-MeSATP (0.03–300 µM) and extracellular ATP marginally less potent (0.03–300 µM), and non-purified ADP considerably less potent (1–1000 µM) [11, 30, 44]. The agonist profile diverged for α,βmeATP and BzATP activity, where two studies [11, 30] reported none but the third [44] showed both synthetic nucleotides were potent agonists. Thus, an agonist potency order of ATP γS ≥ ATP ≥ 2-MeSATP >α,βmeATP ≥ BzATP > ADP may be established. There is one notable difference between these pharmacological studies: the agonist potency across the board was at least tenfold lower in first two studies [11, 30] compared to the third [44]. There is no obvious explanation for this difference, particular since the different potency for the non-hydrolysable ATP γS cannot be explained by an action of ecto-ATPases in respective expression systems.

**Table 2:** Operational and pharmacological profiles of Rp2X5 receptors

rp2X5	[Ref. 11] (1996)	[Ref. 30] (1996)	[Ref. 49] (1998)	[Ref. 31] (1999)	[Ref. 50] (1999)	[Ref. 44] (2002)	[Ref. 13] (2006)	[Ref. 16] (2019)
Responses								
* Expression system*	HEK293 cells	HEK293 cells	HEK293 cells	*X. laevis* oocytes	HEK293 cells	*X. laevis* oocytes	1321N1 cells	HEK293 Cells
* Activation*	Fast (< 500 ms)	Fast (< 200 ms)	Fast (< 200 ms)	Slow (> 2.5 s)	Fast (< 200 ms)	Slow (4.1 ± 0.9 s)	Fast (< 1 s)	Fast (< 1 s)
* Inactivation*	Slow (> 5 s)	Slow (> 2 s)	Slow (> 2 s)	Slow (> 2.5 s)	Slow (> 2 s)	Slow (11.8 ± 3.9 s)	Slow (> 5 s)	Slow (> 5 s)
* Peak ATP response*	< 100 pA (at − 70 mV)	< 400 pA (at − 70 mV)	< 100 pA (at − 40 mV)	< 300 nA (at − 100 mV)	< 100 pA (at − 40 mV)	74 ± 22 nA (at − 95 mV)	3–5 μA (at − 60 mV)	< 100 pA (at − 60 mV)
Agonist EC_50_ values								
* ATP*	15.4 ± 1.2 μM	8 ± 2 μM	^___^	^___^	^___^	0.44 ± 0.05 μM	^___^	27 ± 14 µM
* 2-MeSATP*	20 ± 3 μM	15 ± 4 μM	^___^	^___^	^___^	0.44 ± 0.5 μM	^___^	^___^
* ATPγS*	9.3 ± 1 μM	^___^	^___^	^___^	^___^	0.29 ± 0.17 μM	^___^	^___^
* ADP*	126 ± 21 μM	^___^	^___^	^___^	^___^	3.6 ± 0.3 μM	^___^	^___^
* α,βmeATP*	> 100 μM	> 500 μM	^___^	^___^	^___^	1.1 ± 0.2 μM	^___^	^___^
* BzATP*	^___^	^___^	^___^	^___^	^___^	1.3 ± 0.9 μM	^___^	^___^
Antagonist IC_50_ values								
* PPADS*	2.6 ± 0.3 μM	7 ± 4 μM	^___^	^___^	^___^	0.20 ± 0.02 μM	^___^	^___^
* Suramin*	2.9 ± 0.4 μM	13 ± 7 μM	^___^	^___^	^___^	1.5 ± 0.20 μM	^___^	^___^
* TNP-ATP*	^___^	^___^	^___^	^___^	^___^	0.45 ± 0.18 μM	^___^	^___^
* Reactive blue 2*	^___^	^___^	^___^	^___^	^___^	18.3 ± 0.81 μM	^___^	^___^
* Ip*_*5*_*I*	^___^	^___^	^___^	^___^	^___^	> 30 μM	^___^	^___^
Allosteric modulators								
* Low pH*	^___^	^___^	^___^	^___^	^___^	NAM (pH 7.5–5.5)	^___^	^___^
* Zinc ions*	^___^	^___^	^___^	^___^	^___^	PAM (1–100 μM)	^___^	^___^
* Calcium ions*	^___^	^___^	^___^	^___^	NAM (6.7 ± 1.9 mM)	NAM (time-dependent)	^___^	^___^

In antagonist studies, ATP responses at rP2X5 are blocked by PPADS, Reactive blue 2, suramin, and TNP-ATP, yet are sparingly inhibited by di-inosine pentaphosphate (Ip_5_I) [11, 30, 44]. The potency order of PPADS > TNP-ATP > suramin > RB2 ≫ Ip_5_I for rP2X5 is remarkable for two reasons: (i) Ip_5_I and TNP-ATP are significantly more potent at homomeric rP2X1 at nanomolar concentrations (and may help differentiate rP2X1 from rP2X1/5) [44]; (ii) TNP-ATP, PPADS, and suramin are equally potent at homomeric rP2X5 and heteromeric rP2X1/5 receptors [44, 50]. To date, there are no antagonists which discriminate between rP2X5 and other homomeric and heteromeric P2X receptor subtypes. Additionally, Ip_5_I has not been tested on heteromeric rP2X1/5 receptors.

In terms of allosteric modulation, rP2X5 is affected by the extracellular pH which reduces the potency and efficacy of extracellular ATP at pH5.5 [44]. Extracellular zinc (1–100 µM) potentiated responses to submaximal concentrations of extracellular ATP (300 nM, approximately the EC_50_ concentration), by almost doubling the amplitude of inward currents [44]. Otherwise, extracellular Ca^2+^ causes a time- and concentration-dependent inhibition of ATP responses at rP2X5 [44, 50]. None of the DHP modulators of hP2X5 [18] have been tested on rP2X5.

When taken together, the operational and pharmacological profiles of rP2X5 are reminiscent of rP2X2 (but only if agonist data on α,βmeATP and BzATP are discounted) and the antagonist activities are broadly similar for these two rP2X receptor subtypes [1–3]. In terms of modulators, the actions of extracellular H^+^, Zn^2+^, and Ca^2+^ are not similar for these two P2X receptor subtypes. Furthermore, rP2X2 does not show rundown of successive ATP responses, whereas rundown is profoundly evident for rP2X5. Therefore, rP2X5 bears only a passing resemblance to rP2X2 (as was evident for hP2X2 and hP2X5 in “hP2X5 receptor function”), whereas closer inspection of their respective profiles reveals distinct differences.

### rP2X5 receptor function (2006 onwards)

In 2006, one study yielded the unusual result of large inward currents (at − 60 mV) to extracellular ATP (3 μA at 10 µM, and 5 μA at 1 mM) for N-terminal hexahistidyl-tagged rP2X5 receptors (His-rP2X5) and WT-rP2X5 expressed in oocytes (see Table 2), and with extracellular Ca^2+^ (1 mM) in the superfusate [13]. Other studies using N-terminal His-tagged rP2X1 to identify heteromeric rP2X1/2 assemblies [51] as well as heteromeric rP2X1/4 assemblies [52] failed to report unusually large ATP responses in control experiments with homomeric His-rP2X1 receptors. On a similar note, His-tagged hP2X7 receptors failed to produce unusually large ATP responses although the second phase of P2X7 receptor activation (*I*_2_ response) was inhibited but not the first phase (*I*_1_ response) [53]. For C-terminal tagged rP2X5, neither rP2X5-His6 [31] or rP2X5-HA receptors [49] produced large inward currents (see Table 2). Thus, this singular result with His-rP2X5 and WT-rP2X5 receptors in one study [13] suggests that a naturally occurring substance may allosterically modulate rP2X5 receptors.

In terms of a naturally-occurring substance in the intracellular compartment, PIP_2_ (5µM) was shown to potentiate inwards currents activated by 30 µM ATP by more than 15-fold in inside-out membrane macropatches of oocytes expressing rP2X5 receptors [38].

Paradoxically, ATP responses were neither inhibited by phosphoinositide-depleting wortmannin (10 µM) nor potentiated by PIP_2_ (200 µM) in the recording patch pipette for whole-cell currents evoked by rP2X5 receptors expressed in HEK293 cells, even though the opposite was true for heteromeric P2X1/5 receptors [54]. The proximal C-terminus of the P2X5 subunit failed to bind any phospholipids in a lipid strip assay [54], although adding positive charges by mutating residues 365 and 366 in the C-terminus induced binding to several PIP_n_ compounds in the same lipid assay and also significantly potentiated ATP responses at rP2X5 receptors [40]. At present, the consensus is that rP2X5 does not directly bind PIP_n_ lipids (or perhaps weakly binds to PIP_n_) [8, 55], although this premise fails to explain the observed PIP_2_-potentiation of ATP-activated currents in inside-out membrane patches containing rP2X5 receptors [38].

In terms of a positive allosteric modulator acting on the exterior of rP2X5, the only lead candidate is the DHP compound nimodipine, which is known to potentiate ATP responses at hP2X5-fl [18]. The paucity of functional studies on rP2X5 limits any meaningful comments on positive allosteric modulation of this P2X subtype. However, the outcome of experiments with chimeric h/rP2X5 receptors [10, 16] may raise hope that PAMs can be identified for WT-rP2X5.

### Chimeric human/rat P2X5 receptors

The first generated h/rP2X5 chimera contained the N-terminal portion of hP2X5A (1–318 aa) spliced to the C-terminal portion of rat P2X5 (318–455 aa) [10]. The human peptide provided the intracellular N-terminus domain, the entire TM1 and bulk of the ectodomain (finishing 9 aa upstream of the exon 10 sequence), whereas the rat peptide provided the entire TM2 and the intracellular C-terminus domain (see Fig. 2). When expressed in oocytes, these chimeric h/rP2X5 receptors produced a robust inward current (approximately 1 μA, at − 100 mV) in response to extracellular ATP (100 µM) [10]. However, there was a rapid rundown in the amplitude of successive ATP responses with extracellular Ca^2+^ present (1.8 mM) [10]. At least two conclusions can be drawn: (i) the ectodomains of three hP2X5 subunits are capable of forming functional ATP docking sites and (ii) the assembly of three TM2 regions by rP2X5 subunits is capable of forming a competent ion channel. The observed rundown by the chimera could not be attributed to a particular segment of the chimeric subunit, because rundown is seen with wildtype hP2X5-fl and rP2X5 receptors expressed in oocytes [18, 44].

**Fig. 6:**
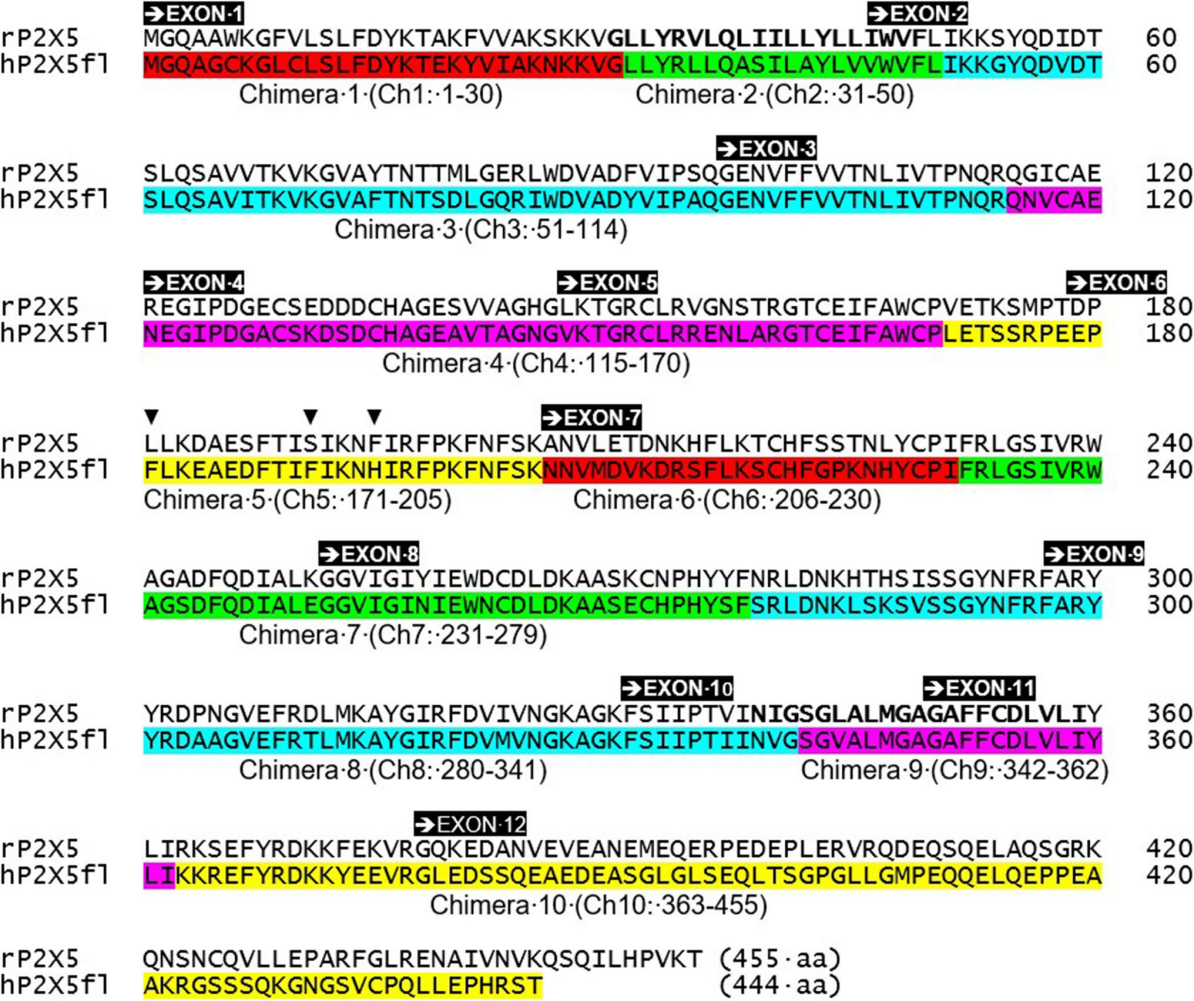
Chimeric constructs using rat P2X5 and human P2X5 subunits. The primary sequence of the rat P2X5 subunit protein (455 aa) and full-length human P2X5 (444 aa). Ten chimeras (Ch 1–Ch 10) were constructed by replacing a succession of peptide sequences in rP2X5 with the corresponding sequences in hP2X5fl. Exon 1–12 sequences are marked by black boxes

A second and more comprehensive series of chimeric h/rP2X5 receptors was more revealing on receptor functionality [16]. A series of 10 chimeras (Ch 1–10) were generated, where a succession of peptide sequences in rP2X5 was replaced with the corresponding sequence from hP2X5-fl (identified in Fig. 6). Insertion of either one of two hP2X5-fl peptide sequences, denoted as Ch 3 (51–114) and Ch 5 (171–205), significantly potentiated ATP responses in chimeric h/rP2X5 receptors, whereas the other eight sequences failed to do so [16]. Except for a reduction in Ch 4, western blots showed no significant difference in the surface expression of other P2X5 chimeras. However, the evoked inwards currents (at − 60 mV) were sizeable for Ch 3 and Ch 5 when activated by extracellular ATP (100 µM): 72 ± 11 pA/pF and 162 ± 21 pA/pF, respectively [16]. The ATP responses for the Ch 5 chimera were similar in amplitude to hP2X5-fl (278 ± 53 pA/pF) and significantly larger than ATP responses at wildtype rP2X5 (2 ± 1 pA/pF).

The P2X5 peptide sequence for hP2X5 at 171–205 (for Ch 5) differs from rP2X5 by 10 residues, and successive replacement of each of these residues produced three mutant rP2X5 receptors that showed a significant gain-of-function: L181F (19 ± 5 pA/pF), S191F (99 ± 2 pA/pF), and F195H (143 ± 20 pA/pF) [16]. As well as an increase in ATP efficacy, the S191F mutant also showed an increase in ATP potency (EC_50_ = 1 ± 0.3 µM, *vs* 27 ± 14 µM for WT). Despite an even greater increase in ATP efficacy, the F195H mutant instead showed a decrease in ATP potency (45 ± 2.0 µM) and evoked currents were marked by their slow rate of activation. The reverse substitution of F191S, but not H195F, in hP2X5-fl reduced the amplitude of ATP responses (*I*_max_ = 39 ± 10 pA/pF *vs* 278 ± 53 pA/pF).

Based on the zebrafish P2X4 crystal [5, 6], homology modelling of rP2X5 receptors indicated a potential interaction in the channel open state between Ser191 and docked ATP [16]. Molecular dynamic simulations further indicated that S191F mutant may cause conformational changes in both left and dorsal flippers in the closed state, with the Phe191 substitution pushing the flexible left flipper away from the ATP-binding pocket. Accordingly, the S191F mutation may increase the opening probability of rP2X5 and promote the transition from closed to open states [16]. Single-channel currents recorded from outside-out patches (− 120 mV, 100 µM ATP) indicated that S191F mutations transition rapidly to the open state, whereas wildtype rP2X5 transitioned more so to a sub-conductance state.

The F195H mutation may cause gain-of-function by enhancing contacts between the polar imidazole ring of His195 and charged residues on the flexible left flipper. Molecular dynamic simulations further indicated that F195H may cause H-bond contacts with several charged residues (Asp282, Thr286, Ser291, and Gly292) of the left flipper when ATP docks with its binding pocket. Consequently, alanine substitution of these identified residues led to reversal of gain-of-function by F195H, whereas adjusting the extracellular pH and protonation of this key histidine residue also regulated the gain-of-function by F195H [16].

For the Ch 3 chimera, the P2X5 peptide sequence for hP2X5 at 51–114 differs from rP2X5 by 10 residues [16]. Successive replacement of these residues only produced one mutant rP2X5 receptors that showed a significant gain-of-function: V67I (29 ± 4 pA/pF). The Val67 residue lies under the left flipper, and its substitution to isoleucine is believed to flex the left flipper. The Val67 residue lies adjacent to above-mentioned Ser291 and Gly392, and it was postulated that the isoleucine mutant may push the Ser291–Gly292 dipeptide away from the ATP-binding pocket after ATP binding. Although this one substitution (V67I) caused gain of function, it still was not as effective as the replacement of the entire Ch3 sequence which caused a gain-of-function of 72 ± 11 pA/pF. This difference indicated that other residues may contribute to the actions of the V67I mutation. Double mutations were not carried out, and it would be interesting to see the impact of V67I with either S191F or F195H.

## Discussion

Even after 25 years, our understanding of the P2X5 receptor is limited. In the first 10 years since cloning rP2X5, investigations on its operational and pharmacological profiles were beset by experimental problems and sometimes by controversial outcomes (particularly on agonist potency and rank order of agonists). Over the next 15 years, there were a relatively low number of studies on P2X5 compared with other P2X receptor subtypes but, nonetheless, a few studies have led to an advancement of our understanding of human and rat P2X5 receptors.

*P2RX5* is not a pseudogene, and categorically does not represent a genomic fossil or junk DNA in the evolution of the human genome [56, 57]. Instead, the *P2RX5* gene encodes a functional P2X subunit for at least a small percentage of humans, principally of African American origin, although this percentage may yet grow as more humans of the same and different ethnic backgrounds are screened for P2X5-fl. Full-length P2X5 is a 444 aa subunit which can assemble efficiently into homomeric P2X5 receptors and, in all probability, also can form heteromeric P2X receptors with other P2X subunits as evidenced with rP2X5 (see Introduction, Human P2X5 receptors and hP2X5 receptor structure).

From work carried out on full-length constructs, hP2X5 is now recognized as a slowly desensitizing, *α*,βmeATP-sensitive P2X channel permeable to sodium, potassium, calcium, and chloride ions. However, too little is known about the physiology of hP2X5-fl to determine what advantages are conferred by the presence of this P2X receptor subtype in human tissues. Conversely, nothing has ever been said of the disadvantages of nonfunctional hP2X5 receptors if there are any. Furthermore, the reported potentiation of hP2X5-fl by one DHP compound (nimodipine) and its inhibition by another (amlodipine) [18] indicates that the physiological and pathophysiological effects of therapeutic drugs such as DHP compounds should be monitored closely in future. The existence of full-length P2X5 in extant *Hominoidea/Hominini* species may provide a useful testbed to elucidate the pharmacological side-effects of such therapeutic drugs.

Rat P2X5 in its full form struggles to function normally, yet a recent study of outstanding breadth has revealed that one of several single-point mutations (V67I, S191F, F195H) can significantly improve its functionality as an ATP-gated ion channel [16]. The authors of this seminal study raised two discussion points: (i) does wildtype rP2X5 exist only to form heteromeric P2X receptors and (ii) do mutations occur in the animal kingdom to enhance the functionality of mutant P2X5 receptors. For the first question, the rP2X5 subunit is promiscuous and may co-assemble with P2X1–4, 6 subunits (but not the P2X7 subunit) based on the outcome of co-immunoprecipitation studies [58]. There is ample scope for rP2X5 to modify the operational profile of many P2X receptor subtypes. For the second question, there are two known variants of zebrafish P2X5 where the operational profile of the second is exceptional for its large biphasic currents to extracellular ATP [32]. The possibility of variants in other species remains to be addressed.

The effects of DHP compounds on hP2X5 raised the possibility that there also may be substances which act as PAMs on the left flipper domain of wildtype rP2X5 and other species isoforms. What we do know from studying heteromeric rP2X1/5 and rP2X2/5 receptors is the apposition of the P2X5 subunit with either P2X1 or P2X2 subunits exerts an allosteric effect on the assembled heteromer such that its functionality is assured and its operational and pharmacological profiles are sufficiently distinct from the homomers otherwise formed by homomeric P2X1 and P2X2 assemblies [1–3]. One past study reported unusually large ATP responses to His-tagged and wildtype rP2X5 receptors [13], raising the possibility that either an unappreciated substance in the perfusate or a substance released from 1231N1 astrocytoma cells in this rP2X5 expression study by can act an allosteric modulator. Thus, our understanding of P2X5 in the animal kingdom may require a thoughtful and thorough reappraisal.

Does it matter if species isoforms of P2X5 work, or are they nonessential? The answer to this question may lie in the discrete distribution of its mRNA and protein in foetal, neonatal and adult tissues [1–3, 10, 11, 14, 15, 30, 31, 44, 59, 60]. A cDNA for hP2X5A was originally isolated from foetal brain, although a stronger mRNA signal by RT-PCR detection was obtained for adult lymphocytes when compared to adult brain [11]. Thus, the primary focus of hP2X5 has been the immune system where mRNA for the more common hP2X5A isoform abounds in peripheral blood T cells, B cells and NK cells, lymphoid tissues, normal progenitor cells, and malignant CD34^+^ progenitor cells [19, 20]. High transcription levels were found in lymphoid tissues (e.g. spleen, tonsils, thymus, and bone marrow), but was not be detected in other tissues (e.g. skin, liver, colon, and small intestine) except for adult brain and skeletal muscle [19, 20]. Any possible utility in knowing this restricted pattern of P2X5 mRNA expression in the immune system lies in promoting the graft-versus-leukaemia reaction following stem cell implantation, where donor-derived cytotoxic T-lymphocytes can eliminate malignant leukaemic cells decorated by a P2X5-linked antigen in the recipient [19, 20]. It is yet to be shown that the expression of full-length P2X5 in lymphoid tissues has any bearing on the incidence of leukaemia in black Americans.

A cDNA for rP2X5 was originally isolated from sympathetic neurons of the coeliac ganglion [11] and spinal cord thereafter [31]. In situ hybridisation experiments failed to show significant levels of P2X5 mRNA in adult rat brain, with the notable exception of the mesencephalic nucleus which contains specialised mechanoreceptive sensory neurons of the trigeminal nerve (cranial nerve V) that serve jaw-closing muscles and monitor tooth pressures when biting down [11]. P2X5 mRNA also was found in primary sensory neurons of the trigeminal ganglia (Gasserian ganglia) and in dorsal root ganglia of the spinal cord [11]. The presence of P2X5 on the sensory nerves from rat skeletal muscles has drawn the most interest [61]. A P2X5-like receptor has been identified as contributory to the sensitisation of an ASIC3-based metaboreceptor, augmenting the responsiveness of ASIC3 channels to small changes in extracellular pH, not only in DiI-labelled sensory neurons innervating skeletal muscle but also in COS and CHO cells expressing recombinant rASIC3 and rP2X5 [61]. It remains to be shown if P2X5-based sensitisation of ASIC3 channels occurs in human skeletal muscle expressing hP2X5-fl, and if DHP-based drugs can further sensitise or inhibit metaboreceptors in human skeletal muscle.

The rP2X5 subunit is 95% identical to the mouse P2X5 subunit (see Fig. 5c), and the operational profile of their P2X5 receptors is considered to be similar [12]. The question arises, what would happen with P2X5 gene deletion in the murine model? There are only a few studies that address the question *P2*×*r5* gene knockout, and none which followed up on the earlier studies on hP2X5 in the immune system or rP2X5 in sensory nerves. However, it has been shown that murine P2X5 is highly expressed during the maturation phase of bone-resorbing osteoclasts (OCs) and that P2X5 gene deletion (*mP2*×*r5*^−/−^) diminishes OC maturation in vitro, and also decreases the rate bone loss in inflamed parietal calvarium (skull) in vivo, yet without affecting normal bone development [62]. The process of murine P2X5-dependent OC maturation involves methylosome protein 50 which physically associates with the C-terminus of the P2X5 receptor [63]. Accordingly, mutant mP2X5 subunits with the TM2 domain and C-terminus deleted (mP2X5Δ268) also results in impaired OC maturation [63]. Expression levels of IL1β, IL6, IL17α, and TNF-sf11 were significantly lower in *P2*×*r5*^−/−^ mice compared to WT mice, and this reduction led to decreased periodontitis (gum disease) and decreased alveolar bone loss compared with WT mice, when challenged by LPS from *Porphyromonas gingivalis* [64]. As pointed out recently [18], the relevance to humans of the effect of P2X5-mediated bone loss in mice remains to be explored.

In summary, P2X5 receptors have been widely ignored for more than 25 years and their roles in physiological and pathophysiological processes have been under appreciated. This current review has attempted to rehabilitate the reputation of mammalian P2X5 receptors and, hopefully, this fresh perspective on the functionality of P2X5 subtype can lead to a better understanding of the human P2X5 receptor in health and disease. The first challenge is to confirm the gene and protein structure of hP2X5-fl cloned from a suitable tissue source, such as human lymphocytes. Thereafter, the pharmacological and operational profiles of hP2X5-fl need to be studied in detail, including the identification of selective antagonists and allosteric modulators. The pharmacological profile of wildtype and mutant rP2X5 receptors also needs further investigation, particularly in terms of allosteric modulators which may help to elucidate the role of rP2X5 (and its orthologue, mP2X5) in physiological processes such as skeletal muscle function at the point of fatigue. Hopefully, interesting times lay ahead for functional P2X5 receptors in the future.

## Data Availability

Data that support this study are available from the corresponding author upon request.
